# Modeling of Cognitive Impairment by Disease Duration in Multiple Sclerosis: A Cross-Sectional Study

**DOI:** 10.1371/journal.pone.0071058

**Published:** 2013-08-01

**Authors:** Anat Achiron, Joab Chapman, David Magalashvili, Mark Dolev, Mor Lavie, Eran Bercovich, Michael Polliack, Glen M. Doniger, Yael Stern, Olga Khilkevich, Shay Menascu, Gil Hararai, Micharel Gurevich, Yoram Barak

**Affiliations:** 1 Multiple Sclerosis Center, Sheba Medical Center, Tel-Hashomer, Israel; 2 Neurology Department, Sheba Medical Center, Tel-Hashomer, Israel; 3 Sackler School of Medicine, Tel-Aviv University, Tel Aviv, Israel; 4 NeuroTrax Corporation, Ono Academic College, Kiryat Ono, Israel; 5 MediStat Ltd., Ramat Hachayal, Tel-Aviv, Israel; 6 Abarbanel Mental Health Center, Bat-Yam, Israel; University of Düsseldorf, Germany

## Abstract

**Background/Aims:**

Large-scale population studies measuring rates and dynamics of cognitive decline in multiple sclerosis (MS) are lacking. In the current cross-sectional study we evaluated the patterns of cognitive impairment in MS patients with disease duration of up to 30 years.

**Methods:**

1,500 patients with MS were assessed by a computerized cognitive battery measuring verbal and non-verbal memory, executive function, visual spatial perception, verbal function, attention, information processing speed and motor skills. Cognitive impairment was defined as below one standard deviation (SD) and severe cognitive impairment as below 2SD for age and education matched healthy population norms.

**Results:**

Cognitive performance in our cohort was poorer than healthy population norms. The most frequently impaired domains were information processing speed and executive function. MS patients with secondary-progressive disease course performed poorly compared with clinically isolated syndrome, relapsing-remitting and primary progressive MS patients. By the fifth year from disease onset, 20.9% of patients performed below the 1SD cutoff for impairment, p = 0.005, and 6.0% performed below the 2SD cutoff for severe cognitive impairment, p = 0.002. By 10 years from onset 29.3% and 9.0% of patients performed below the 1SD and 2SD cutoffs, respectively, p = 0.0001. Regression modeling suggested that cognitive impairment may precede MS onset by 1.2 years.

**Conclusions:**

The rates of cognitive impairment in this large sample of MS patients were lower than previously reported and severe cognitive impairment was evident only in a relatively small group of patients. Cognitive impairment differed significantly from expected normal distribution only at five years from onset, suggesting the existence of a therapeutic window during which patients may benefit from interventions to maintain cognitive health.

## Introduction

Cognitive impairment is a major predicament in multiple sclerosis (MS) and adversely affects patients’ quality of life. Cognitive decline may appear early in the disease process and has been reported even at disease onset [1]. Prevalence of cognitive impairment in MS has been estimated to occur in 20% to 65% of patients [2], [3]. This wide range is probably related to variation in the disease subtypes, disease duration and level of disability of the MS population studied, as well as to differences in the cognitive assessment scales, procedures and tools used. Moreover, variation in the tests’ cutoff adopted as indicative of cognitive impairment and criteria for inclusion of patients with significant cognitive decline or motor disability may also account for the wide range of prevalence estimates across studies [4].

The influence of disease duration on cognitive functioning in MS remains controversial. While some studies report no correlation between disease duration and cognition [5], [6], a trend for higher frequency of poor cognitive performance was reported in patients with longer disease duration [7]. Investigations of the natural history of cognitive function along the disease course are frequently confounded by small sample sizes, inadequate control for practice effects, high drop-out rates and brief retest intervals [8]–[11].

The cognitive functions most commonly impaired in MS patients are verbal and visual memory, including difficulties in learning and forgetfulness, especially recall of recently learned information; memory impairment has been reported in 22% to 31% of patients [12]. Impairment in the information processing domain, including increased distractibility and slowing of mental functioning, has also been commonly reported with prevalence of 22% to 25% in MS patients [6], [13], [14]. We have previously shown prevalent cognitive impairment in MS patients evaluated during the onset of the first neurological symptomatology, wherein verbal abilities and attention span were the most frequently affected [15]. Additionally, in a group of 150 relapsing-remitting MS patients with disease duration up to 15 years, we characterized the timeframe of appearance of particular types of cognitive impairment and demonstrated that verbal fluency and verbal memory were first affected followed by a decrease in visuospatial learning, delayed recall and then by a decrease in attention and information processing speed [16].

This significant cognitive involvement in MS requires a comprehensive and valid assessment that is suitable for patients’ follow-up over-time. Moreover, routine evaluation of cognitive change may be useful for helping MS patients manage activities of daily living that are adversely affected by cognitive decline, and early detection of cognitive impairment may be used to target patients for specific therapeutic strategies.

A number of screening batteries have been developed for the assessment of cognition in MS patients [6], [17], [18]. In the last decade the use of computerized cognitive assessment that provides neuropsychologists and cognitive experts with a rapid, standardized, and precise screening assessment of cognitive performance has emerged [19]–[22]. These computerized assessments permit the measurement not only of accuracy of response, but also of the time needed to make that response, thus adding the novel dimension of precise response-time measurement to the cognitive paradigm. Nevertheless computerized assessment must be used responsibly, with attention to whether the test will be useful, accurate, and appropriate in the intended setting [19], [22].

In MS patients we have shown that the computerized MindStreams Global Assessment Battery (GAB; referred to in our earlier work as the MindStreams Computerized Cognitive Battery, MCCB) has good discriminant validity for memory, information processing, executive function, attention and motor skills domains, and good construct validity relative to the NSBMS, though construct validity of the GAB visual spatial, verbal function and motor skills index scores could not be evaluated in the absence of NSBMS tests in comparable cognitive domains [23]. GAB performance has also been reported to be related to MS disease severity and duration, quality of life, patient perceived deficit, and immunomodulatory (IMD) treatment [24]–[27].

In the current study, we report on a large cross-sectional evaluation of 1,500 consecutive MS patients’ cognitive performance using the GAB to model the relationship between disease duration and cognitive performance.

## Methods

### Study Population and Setting

Data were collected from the Sheba MS computerized database, a population-based registry documenting demographic and clinical data of all MS patients followed at Sheba Medical Center, Tel-Hashomer, Israel, since 1 January 1995. The Multiple Sclerosis Center at Sheba Medical Center was established to provide long-term multidisciplinary care and treatment for patients diagnosed with MS from referral areas all over the country and is currently following and treating 2156 patients out of a total of ∼4000 MS patients in Israel. Since the establishment of the MS Center, an electronic record-keeping system has been used to archive patients’ demographic, clinical and imaging data and is updated by the Center’s neurologists during each clinic visit. Patients’ computerized files include demographic data, medical history, family history, cognitive, electrophysiological and neuroimaging tests results, and dates of steroids, immunomodulatory and other drugs treatments. At each patient’s visit to the clinic, a complete neurological examination is performed and an Expanded Disability Status Scale (EDSS) score is assigned and recorded. Additional evaluations like cognitive assessment, MRI imaging, blood tests, evoked potentials, treatment response, and gene expression data are recorded for each patient as well. Cognitive assessment is part of the routine evaluation of patients at the MS Center and neurological examination with EDSS score is performed within up to 3 months interval. The integrity of the data registry is evaluated by a computerized logic-algorithm-questioning process to identify data entry errors.

The present study describes the cognitive data of 1500 MS patients from the entire cohort of 2156 patients. Key eligibility criteria were an age of 18 to 65 years, a diagnosis of MS, according to the revised McDonald criteria [28], and an interval of up to 3 months between the cognitive assessment and the EDSS examination. Key exclusion criteria were acute MS relapse, corticosteroid treatment within 30 days before the cognitive assessment, other significant neurologic or psychiatric illness and alcohol or drug abuse. Data from patients with clinically significant major depression or anxiety as assessed by the Hamilton questionnaires for depression and anxiety were excluded (N = 171). Each patient’s record was indexed by an anonymous code number to ensure confidentiality during statistical analyses. For patients with multiple cognitive assessments, data for one visit was selected at random by computerized software so that each patient is represented only once in the study dataset. The study was approved by the Sheba Hospital Research Ethics Committee (Ethics Ref: 5596-08/141210).

### Cognitive Assessment

Cognitive assessment was performed using the MindStreams GAB (NeuroTrax Corp., Bellaire, TX, USA), a 45-minute computerized battery designed to assess cognitive function in healthy to mildly impaired individuals using custom software installed on a standard computer to measure accuracy and response times. For any test that measured response time, patients were instructed to respond as quickly as possible. The GAB produces 65 outcome parameters from 10 tests that cover the following cognitive domains: verbal and non-verbal memory, executive function, visual spatial processing, verbal function, attention, information processing speed and motor skills. A detailed description of the test battery and Index scores explanation that designate the cognitive domains tested, provide a short test description and identify the outcome parameters is demonstrated in Table 1. Outcome parameters (accuracy, response time) are normalized for age and education according to stratifications of a normative database (N = 1569) of cognitively healthy subjects. This normative sample included cognitively healthy participants in controlled research studies (e.g., control participants in discriminant validity or interventional studies). Details are available here: http://www1.mindstreamshealth.com/docs/norms_guide.pdf.

**Table 1:** MindStreams Global Assessment Battery (GAB): Description of cognitive domains tested and outcome parameters obtained.

GAB Test	Cognitive Domains Tested	Test Description	Outcome Parameters
GO-NOGO RESPONSE INHIBITION	Executive Function, Attention	Timed continuous performance test during which responses are made to large colored stimuli that are any color but red.	AccuracyAverage Response TimeResponse Time Standard DeviationErrors of CommissionErrors of OmissionResponse Time for Errors of Commission
VERBAL MEMORY	Memory	Ten pairs of words (the study set) are presented, followed by a recognition test in which one member (the target) of a previously presented pair appears together with a list of four candidates for the other member of the pair. There are four immediate repetitions and one delayed repetition after 10 minutes.	*Immediate Recognition*Accuracy, Repetition 1 to 4Accuracy, Repetitions 1–4*Delayed Recognition*Accuracy
NON-VERBAL MEMORY	Memory	This is similar to the test of verbal memory, except that geometric figures are used instead of words.	*Immediate Recognition*Accuracy, Repetition 1 to 4Accuracy, Repetitions 1–4*Delayed Recognition*Accuracy
PROBLEM SOLVING	Executive Function	Pictorial puzzles of gradually increasing difficulty are presented. Each puzzle consists of a 2×2 array containing three black-and-white geometric forms with a certain spatial relationship among them and a missing form. Participants must choose the best fit for the fourth (missing) form from among six possible alternatives.	Accuracy (Non-Verbal IQ)
STROOP INTERFERENCE	Executive Function, Attention	Timed test of response inhibition and set shifting. For example, in the ‘No Interference [Meaning]’ phase, the task is to choose the color named by a word presented in white letter-color. In the final (‘Interference’) phase, participants choose the letter-color of a word that names a different color.	*No Interference: Letter Color*AccuracyAverage Response TimeResponse Time Standard Deviation*No Interference: Word Meaning*AccuracyAverage Response TimeResponse Time Standard Deviation*Interference: Letter Color vs. Word Meaning*AccuracyAverage Response TimeResponse Time Standard Deviation
FINGER TAPPING	Motor Skills	Participants must tap on the mouse button with their dominant hand.	Inter-Tap IntervalTap Interval Standard Deviation
CATCH GAME	Executive Function, Motor Skills	A test of motor planning requiring hand-eye coordination and rapid responses. Subjects “catch” a “falling object” by moving a “paddle” horizontally on the computer screen so that it can be positioned directly in the path of the falling object.	Time to Make 1st MoveTime to Make 1st Move Standard DeviationAverage Direction Changes Per TrialAverage Error For Missed CatchesTotal Score
STAGED INFORMATION PROCESSING SPEED	Attention, Information Processing Speed	This test comprises three levels of information processing load: single digits, two-digit arithmetic problems (e.g., 5-1), and three-digit arithmetic problems (e.g., 3+2-1). For each of the three levels, stimuli are presented at three different fixed rates, incrementally increasing as testing continues. Participants are instructed to respond as quickly as possible by pressing the left mouse button if the digit or result is less than or equal to 4 and the right mouse button if it is greater than 4.	*SINGLE DIGIT* *Slow Speed, Medium, Fast Speed*AccuracyAverage Response TimeResponse Time Standard Deviation*TWO-DIGIT ARITHMETIC* *Slow Speed, Medium, Fast Speed*AccuracyAverage Response TimeResponse Time Standard DeviationTHREE-DIGIT ARITHMETIC*Slow Speed, Medium, Fast Speed*AccuracyAverage Response TimeResponse Time Standard Deviation
VERBAL FUNCTION	Verbal Function	Pictures of common objects are presented; in the first phase, the word that best rhymes with the name of the object must be selected from among four choices; in the second phase, the name of the picture must be selected.	*Rhyming*Accuracy, High and Low Familiarity*Naming*Accuracy, High and Low Familiarity
VISUAL SPATIAL PROCESSING	Visual Spatial	Computer-generated scenes containing a red pillar are presented. Participants must select the view of the scene from the vantage point of the red pillar.	Accuracy

Index scores explanation for GAB cognitive tests.

1. MEMORY: mean accuracies for learning and delayed recognition phases of Verbal and Non-Verbal Memory tests.

2. EXECUTIVE FUNCTION: composite scores (accuracy divided by average response time) for interference phase of the Stroop test and Go-NoGo test, mean weighted accuracy for Catch Game.

3. VISUAL SPATIAL: mean accuracy for Visual Spatial Processing test.

4. VERBAL: weighted accuracy for verbal rhyming test (part of Verbal Function test).

5. ATTENTION: mean response times for the Go-NoGo test and a no interference phase of the Stroop test, mean response time for a low-load stage of Staged Information Processing test, mean standard deviation of response time for the Go-NoGo test, mean accuracy for a medium-load stage of Information Processing test.

6. INFORMATION PROCESSING: composite scores (accuracy divided by average response time) for various low- and medium-load stages of the Staged Information Processing test.

7. MOTOR SKILLS: mean time until first move for Catch Game, mean inter-tap interval and standard deviation of inter-tap interval for Finger Tapping test.

To permit averaging performance across different types of outcome parameters (e.g. accuracy, response time), each outcome parameter was normalized and fit to an IQ-like scale (mean: 100, SD: 15) stratified by age and education. Sets of normalized outcome parameters are averaged to produce the domain index scores, which are computed to summarize performance in each cognitive domain, Table 1. A Global Cognitive Score (GCS) is computed as the average of the index scores. We defined a value of 85 (i.e., -1SD) as the cutoff for cognitive impairment and a value of 70 (i.e., -2SD) as the cutoff for severe cognitive impairment. GAB has been validated in cognitively healthy individuals, in those with mild cognitive impairment, and in MS patients, and has been found to have good test-retest reliability and construct validity relative to paper-based tests [27], [29]–[31]. Specifically, in MS patients, GAB showed discriminant validity for memory, information processing, executive function, attention and motor skills domains [23]. We have correlated between the cognitive performance of the GAB and the frequently used NSBMS (Neuropsychological Screening Battery for Multiple Sclerosis,) in a group of 58 MS patients (mean ± SD age 41.5±10.0 years, disease duration 9.1±7.4 years and neurologic disability by the EDSS score 2.6±1.8). The NSBMS was administered in a single 30-min testing session. The battery includes short- and long-term verbal (Selective Reminding Test) and spatial (7/24 Spatial Recall Test) memory, attention and information processing speed (Paced Auditory Serial Addition Test; PASAT), and verbal fluency (Word List generation), [6]. GAB Memory showed significant correlation with outcomes from the NSBMS Selective Reminding test and with the NSBMS 7/24 Spatial Recall test. GAB Executive Function showed good correlation with outcomes from the NSBMS Word List Generation test. GAB Attention index showed a higher correlation with PASAT2 than with PASAT3 performance, consistent with the greater attention demands of the harder versus easier version. GAB Information Processing showed moderate correlations with the PASAT test. Gab Visual Spatial, Verbal Function and Motor Skills could not be evaluated as the NSBMS does not include tests in comparable cognitive domains, Table S1. To minimize intra-session practice effects, GAB incorporates test designs that are easy to master, as well as a computer orientation and practice sessions performed prior to individual tests to familiarize the patient with test mechanics. Since 2004, the GAB is routinely performed by MS patients followed at our center as a screening tool for profiling cognitive function and detection of cognitive impairment.

### Statistical Analysis

Statistical analysis was performed using SAS® version 9.1 (SAS Institute, Cary, North Carolina). Analyses included descriptive statistics for demographic, clinical and cognitive data. The chi-square test was applied to test for variation in frequencies of categorical variables among MS subtypes. Principal component analysis (PCA) was used to identify differential cognitive profiles among MS subtypes. PCA is an algorithmic approach commonly used in high dimensional data, where a set of observations are converted into orthogonal principal components that are linear combinations of the original variables. PCA used the correlation matrix method to standardize the data to a mean of zero and a standard deviation of one. This adjustment is performed during the computation and does not modify the original data. The correlation matrix method is used with widely differing variables or when variables are measured in different units. The eigenvectors are scaled using normalization methods. The first principal component describes the dimension that displays the greatest variation in the dataset; the second principal component describes the dimension that displays the second greatest variation, etc. The first two or three principal components usually describe the majority of the variation seen in the entire dataset. PCA allows the user to view three-dimensional plots that show the relative similarity of individuals along principal components in an unbiased fashion. For each patient‘s cognitive dataset (comprised of the seven GAB index scores), an orthogonal transformation was performed to compare the cognitive parameters across disease subtypes.

Analysis of variance (ANOVA) was used to test for differences in cognitive variables among MS subtypes, to evaluate the source of variation of demographic and clinical variables (e.g., age, gender, disease duration, neurologic disability as measured by EDSS score and IMD treatment) on cognitive performance, and to detect differences in cognitive performance among disease duration subgroups by one- and five-year intervals, up to 30 years. Multiple linear regression was applied to test the relationship between GCS as the dependent variable and demographic and clinical independent variables (e.g., age, gender, disease duration, disease type, neurologic disability, and IMD treatment). Odds ratios were calculated by logistic regression model to predict GCS scores less than 85 and less than 70 (-1SD and -2SD cutoffs, respectively). Both linear and logistic regressions were performed as the former gives information about the GCS as a continuous variable and the latter gives information about it when adopting a particular cutoff (i.e., -1SD or −-2SD). For each index score and the GCS, graphs were generated showing the 95% confidence interval about the mean over the range of disease durations. Frequency of cognitive impairment at the -1SD and -2SD cutoffs was calculated for the GCS, and chi-square was used to identify significant differences between the observed and expected values and to compare these frequencies across disease duration subgroups. All tests were two-tailed, and a p-value of 0.05 or less was considered statistically significant.

## Results

### Patient Descriptive Data

Demographic, clinical and cognitive data for all 1,500 MS patients included in the study, and separately for each MS disease subtype, are shown in Table 2.

**Table 2:** Descriptive data for 1500 MS patients.

Characteristics	CIS	RRMS	SPMS	PPMS	All	
n	200	1173	100	27	1500	
Females, n (%)	125 (62.5)	812 (69.2)	54 (54)	12 (44.4)	1003 (66.9)	
Males, n (%)	75 (37.5)	361 (30.8)	46 (46)	15 (55.6)	497 (33.1)	
Age at onset, yrs	33.5±0.7	30.1±0.3	30.9±1.1	45.4±2.1	30.9±0.3	
Age at cognitive assessment, yrs						
Mean±SE	36.8±0.8	39.9±0.3	51.4±1.1	54.2±2.0	40.5±0.3	
Median	35	39	51.5	57	40	
Disease duration, yrs						
Mean±SE	3.2±0.4	9.8±0.2	20.5±1.1	8.9±1.0	9.7±0.2	
Median	1	8.2	21.0	7.6	7.6	
EDSS						
Mean±SE	1.7±0.1	2.7±0.7	6.0±0.7	4.5±0.3	2.8±0.1	
Median	1.5	2.0	6.0	4.5	2.0	
IMD treated, n (%)	34 (17)	849 (72.4)	94 (94)	10 (37)	987 (65.8)	
**Cognitive performance**						p
Mean±SE						
Median						
Global Cognitive Score	92.1±0.8	90.6±0.4	81.3±1.5	90.5±2.3	90.2±0.3	P, P^a^,P^b^ = <0.0001
	94	93	83	94	93	P^c^ = 0.0011
Information Processing Speed	89.3±1.0	89.0±0.5	79.5±2.1	87.0±2.6	88.5±0.4	P, P^a^,P^b^ = <0.0001
	90	89	77	87	89	P^c^ = 0.0362
Attention	92.3±0.9	90.5±0.5	79.8±1.9	88.7±2.9	92.1±0.8	P, P^a^,P^b^ = <0.0001
	94	94	82.5	94	91	P^c^ = 0.0089
Verbal Function	92.2±1.6	92.2±0.6	85.3±2.7	92.3±4.1	91.8±0.6	P = 0.25, P^a^ = 0.111
	100	98	93	99	99	P^b^ = 0.0037, P^c^ = NS
Visual Spatial Perception	94.3±1.3	93.8±0.5	91.3±1.7	98.3±3.2	93.8±0.5	NS
	98	95	92	96	95	
Executive Function	93.5±0.9	90.8±0.4	81.6±1.6	89.6±2.5	90.5±0.4	P, P^a^,P^b^ = <0.0001
	95	92	82	90	92	P^c^ = 0.0127
Memory	92.6±1.2	90.7±0.5	79.5±2.2	92.7±3.2	90.3±0.5	P, P^a^,P^b^ = <0.0001
	99	97	84	99	97	P^c^ = 0.0011
Motor Skills	92.8±1.0	90.5±0.5	82.3±2.2	89.6±2.9	90.4±0.4	P, P^a^,P^b^ = <0.0001
	96	94	84	91	94	P^c^ = 0.045

P = p by ANOVA after Bonferroni correction for group comparison; p^a^ = p between CIS and SPMS; p^b^ = p between RRMS and SPMS; p^c^ = p between PPMS and SPMS. N = number; SE = standard error of the mean; CIS = clinically isolated syndrome; RRMS = relapsing-remitting multiple sclerosis; SPMS = secondary progressive multiple sclerosis; PPMS = primary progressive multiple sclerosis; EDSS- Expanded disability status scale; IMD – Immunomodulatory drugs.

### Cognitive Performance

Cognitive performance in MS patients was consistently below the normalized mean (of 100) for a cognitively healthy sample of similar age and education. MS patients with secondary-progressive (SPMS, N = 100) disease course performed most poorly, with lower scores (mean+SE GCS 81.3±1.5) than patients with clinically isolated syndrome (CIS, N = 200, mean+SE GCS 92.1±0.5), p<0.0001; When compared to relapsing-remitting multiple sclerosis (RRMS, N = 1173) and primary progressive multiple sclerosis (PPMS, N = 27), SPMS patients again performed poorly (p<0.0001). It is of interest to note that apart from the GCS, SPMS patients’ performance was below that of all other disease subtypes in all but one cognitive domain (visual spatial). No significant differences in GCS and cognitive index scores were found when comparing CIS, RRMS and PPMS patients, Table 2.

### Clustering MS Disease Subtypes

**Figure 1:**
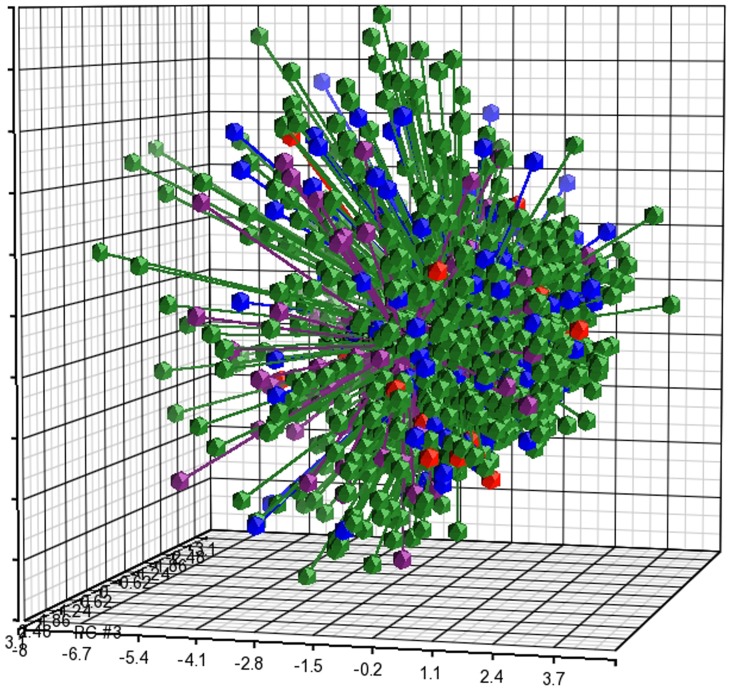
PCA of cognitive performance in MS. PCA of cognitive performance in CIS patients (N = 187, blue dots), RRMS patients (N = 1173, green dots), SPMS patients (N = 100, purple dots) and PPMS patients (N = 27, red dots) demonstrates that cognitive performance in the different disease types are clustered together with a probability of 76.8%. Each dot represents how the sample (subject) is localized in space on the basis of its cognitive performance. The distance between any pair of points is related to the similarity between the two observations in high-dimensional (3D) space.

Evaluation of differential cognitive profiles among MS subtypes by PCA analysis of the three first principal components PC1, PC2 and PC3 representing the linear combinations of the seven GAB index scores plotted to show the proportion of variance explained by each component, demonstrated that the scores were closely clustered for CIS, RRMS, SPMS and PPMS patients, and that the various MS disease subtypes have highly similar cognitive patterns with a reliability of 76.8% for covering the total variance in the data, Fig. 1.

Variation in cognitive performance may be attributable to age, gender, disease duration, IMD treatment and neurological disability. Source of variation analysis revealed that neurological disability had the highest relative contribution followed by age, gender and disease duration, Fig. S1.

Multiple linear regression analysis demonstrated that neurological disability (EDSS score), age of onset and disease duration significantly predicted cognitive performance; Logistic regression analyses for GCS less than 85 and less than 70 (-1SD and -2SD cutoffs) demonstrated that EDSS score followed by age of onset, significantly affected GCS score less than 85, while GCS score less than 70 was additionally affected by disease duration, Table S2, Table S3.

For the entire cohort, the prevalence of impairment (percent patients scoring <85, <1SD) in each cognitive domain was (in descending order): Information processing speed (36.9%), executive function (31.4%), motor skills (28.5%), visual spatial perception (28.2%), memory (27.6%), attention (27.4%) and verbal function (23.0%).

### Cognitive Performance in Relation to Disease Duration

**Figure 2:**
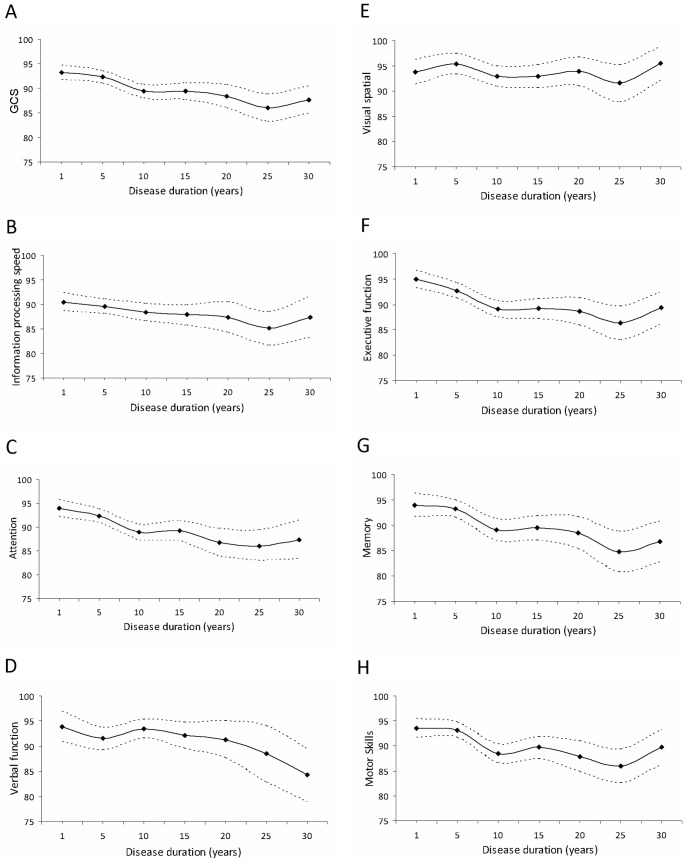
Cognitive performance as a function of MS disease duration. Cognitive performance for MS patients with disease durations of 1 to 30 years (5-year intervals) with 95% confidence intervals for GCS (A) and individual cognitive domains (B–H), N = 1500.

To further explore the relationship between disease duration and cognitive decline, we analyzed cognitive performance in patients with disease durations of 1, 5, 10, 15, 20, 25 and 30 years. Fig. 2 shows plots of mean cognitive performance with 95% confidence interval by disease duration for GCS (A) and the cognitive domains (B–H). The findings demonstrate a significant decrease in all cognitive domains with disease progression by the fifth year from disease onset. Interestingly, for the two domains most often impaired, sharper decline was observed in executive function (B) as compared with information processing speed (F), which was already relatively poorer at onset.

**Figure 3:**
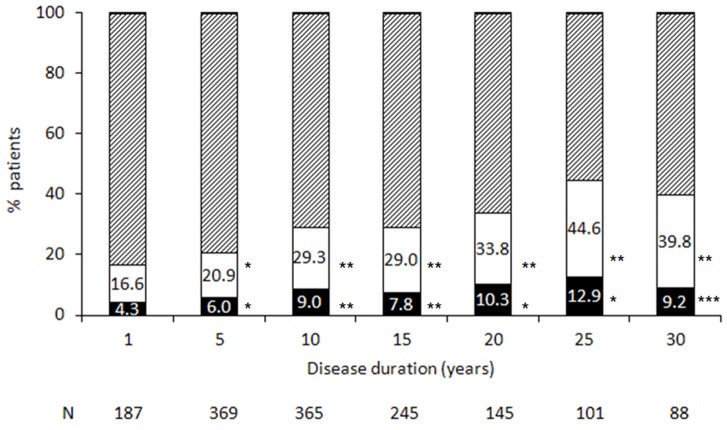
Percent of MS patients with cognitive impairment by disease duration. The percent of patients with impairment in GCS at a cutoff of 85 (1SD below the normalized mean, white bars) and at a cutoff of 70 (2SD below the normalized mean, black bars) is presented by disease duration. The dashed bars represent the percent of patients with GCS ≥85. N = number of patients; *p≤0.005; **p<0.001; ***p<0.05.

The percent of patients with impairment in GCS (score less than 85, 1SD below the mean), and with severe impairment (score less than 70, 2SD below the mean) for subgroups of disease duration, is shown in Figure 3. By the fifth year from disease onset, 20.9% of patients had a GCS below the -1SD cutoff for impairment, p = 0.005, and 6.0% had a score below the -2SD cutoff for severe impairment, p = 0.0021. Similarly, by 10 years from onset, 29.3% of patients had a GCS below the -1SD cutoff for impairment, p = 0.0001, and 9.0% performed below -2SD cutoff for severe impairment, p = 0.0001; a significant decrease in GCS was found up to 30 years from onset. As expected, progressively poorer performance for patient with longer disease duration was evident for all cognitive domains, and the percent of patients with cognitive impairment (by either SD cutoff) was significantly higher than expected from five to 30 years post-onset, Table S4.

### Cognitive Impairment within the First Year from MS Onset

**Figure 4:**
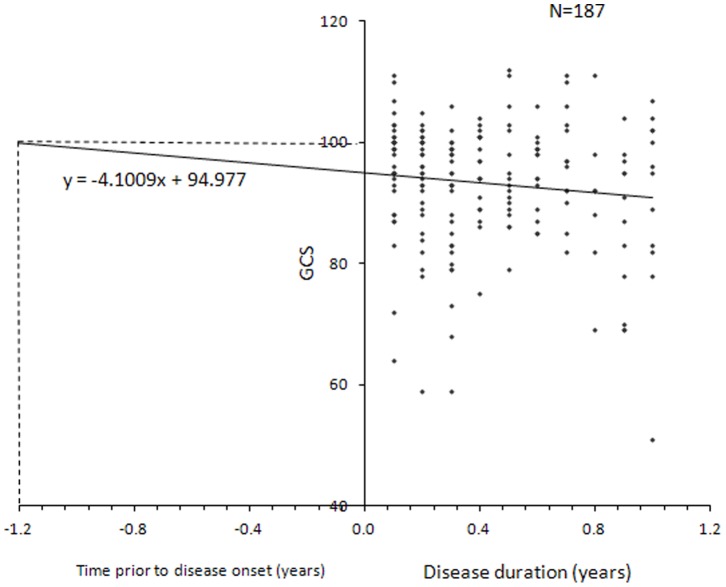
Cognitive performance by GCS within the first year from MS onset, N = 187.

Analysis of cognitive performance by disease duration in MS patients within the first year of MS onset (N = 187) is shown in Figure 4. Overall cognitive performance was below average with progressively poorer performance for patients with longer disease duration. Plotting a regression line for GCS performance of these patients yielded the equation y = −4.1009x+94.977, with an error estimate of 2.7 months. Extrapolating from our disease duration data, we estimate that cognitive performance would have been consistent with cognitive health (i.e., a score of 100) 1.2 years prior to disease onset. This suggests that the pathogenic process associated with cognitive decline begins operating prior to the onset of the clinical disease symptomatology.

## Discussion

The current study evaluated cognitive performance in a large cohort of MS patients with disease durations of up to 55 years. To the best of our knowledge, this is the first study to provide estimates of cognitive impairment in MS over such a range of disease durations. The findings highlight the fact that cognitive impairment in MS is a complex, dynamic process over time, involving multiple cognitive domains and their corresponding brain structures [32], [33]. Our first finding is that cognitive performance in MS patients was below average for age and education matched norms. MS patients with secondary-progressive disease course performed most poorly, with lower cognitive scores than CIS, RRMS and PPMS patients in all but the visual spatial domain. Notably, SPMS patients in the present cohort had mean disease duration twice as long as either RRMS or PPMS patients. We thus reasoned that disease duration may be a major contributing factor to cognitive decline in MS. Previous studies have assessed long-term outcomes, but only for selected cognitive parameters and often without comparison among disease subtypes [34]–[36]. In the present study the pattern of cognitive performance was similar in CIS, RRMS, SPMS and PPMS patients. This suggests that at least from a cognitive point of view, the ongoing demyelinating pathological process similarly affects various brain connections in the early or late phase of the disease. Indeed, although grey matter loss had been reported in SPMS patients, we have recently found cortical atrophy to also occur in the early stage of RRMS [33].

Prevalence of cognitive impairment was significantly greater than expected in patients with disease duration longer than five years. The observed percent of patients performing below the 1SD cutoff for cognitive impairment was 20.9% for disease duration of five years and 29.3% for disease duration of 10 years, while only 6.0% and 9.0% of patients with disease durations of 5 and 10 years, respectively, performed below the 2SD cutoff for severe cognitive impairment. These rates are lower than those previously reported in the literature [6], [37], and indicate that severe cognitive impairment as measured by the GAB is evident in a relatively small group of patients. Moreover, the fact that cognitive impairment was significant only at disease duration greater than five years suggests the existence of an early therapeutic window during which IMD treatment to decrease disease activity and the acquisition of additional neurological disability may involve a cognitive benefit. In this respect, early treatment may also prevent cognitive decline, especially given our finding that neurological disability was the most significant contributor to variation in cognitive performance, implying that patients with active disease who acquire greater disability within short disease duration are also disposed to develop early cognitive decline.

We believe the robustness of our findings is attributable to the large, heterogeneous sample and the breadth and sensitivity of the cognitive assessment tool we employed. Our sample is characterized by great variability, with four disease subtypes and a wide range of disease durations represented. Previous studies in smaller groups of MS patients have similarly shown a differential pattern of cognitive decline with disease progression, involving cognitive functions related to information processing speed, motor skills, episodic memory, attention and visuospatial short-term memory [12], [38], [39].

In the present cohort the majority of patients (65.8%) were exposed to IMD. We were not able to perform analyses related to specific effects of treatment due to variability in compounds prescribed and treatment duration. Nonetheless, our findings suggest that cognitive decline is an integral part of the disease and should be evaluated in studies of IMD treatments.

Finally, we modeled the effect of cognitive impairment in very early MS by analyzing cognitive performance in patients within the first year of clinical presentation. This analysis revealed two important findings. The first is progressively poorer performance for patients, even within this brief period, and the second is that overall cognitive performance at these brief disease durations was below average for age and education. Patients in the early stage of MS are more sensitive to IMD treatments compared with patients with advanced disease [40], [41], thus early treatment may also favorably influence the progression of cognitive decline [42]–[44]. Moreover, our linear regression estimate suggests that cognitive decline associated with MS begun 1.2 years prior to the appearance of clinical symptomatology. This result is in accordance with reports on demyelinating lesions observed in asymptomatic healthy subjects years before the appearance of clinical symptomatology [45], [46], and with our recent study demonstrating a silent MS trait associated with suppressed expression of the nuclear receptor network and inhibited apoptosis of activated T-cells operating in the pre-disease stage of MS [47]. Similarly, in support of a latent, progressive pathogenic process, cognitive performance in subjects with radiologically isolated syndrome was significantly lower as compared with healthy subjects [48]. Our data showing progressive decline over the years suggest the suitability of linear rather than abrupt cognitive decline preceding clinical diagnosis. This is further supported by findings in newly diagnosed MS patients where linear dynamics over time were demonstrated in several cognitive domains [9].

In conclusion, the present study reports rates and patterns of cognitive impairment in a large cohort of MS patients for a broad range of disease durations. Our modeling may help improve MS care by highlighting the need for repeated cognitive assessment as well as interventions tailored to the magnitude and profile of cognitive impairment as the disease progresses.

## Supporting Information

Figure S1 **Variation in cognitive performance.**
(TIF)

Table S1 **Correlations between GAB and NSBMS in a group of 58 MS patients.**
(DOCX)

Table S2 **Multiple linear regression analysis: GCS as the dependent variable.**
(DOC)

Table S3 **Odds ratio estimates by logistic regression.**
(DOC)

Table S4 **% patients with cognitive impairment at the cutoff level of -1SD and -2SD by disease duration.**
(DOC)
