# Supplementary figures and images for: Modeling of Cognitive Impairment by Disease Duration in Multiple Sclerosis: A Cross-Sectional Study
Source: PLoS One. 2013 Aug 1;8(8):e71058. doi: 10.1371/journal.pone.0071058 (PMC3731335; doi:10.1371/journal.pone.0071058)

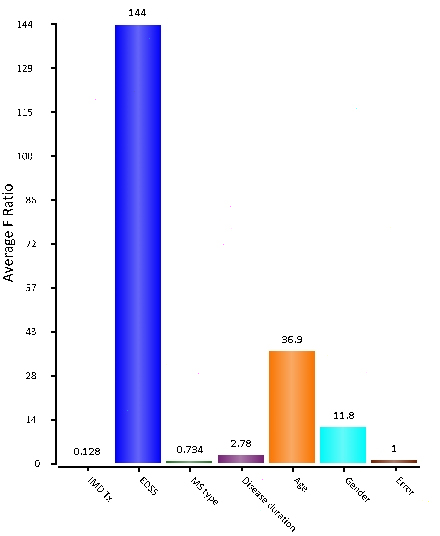

Supplement: Figure S1 — Variation in cognitive performance. (TIF) [file pone.0071058.s001.tif]
