# Supplementary material for: Modeling of Cognitive Impairment by Disease Duration in Multiple Sclerosis: A Cross-Sectional Study
Source: PLoS One. 2013 Aug 1;8(8):e71058. doi: 10.1371/journal.pone.0071058 (PMC3731335; doi:10.1371/journal.pone.0071058)
Supplement: Table S1 — Correlations between GAB and NSBMS in a group of 58 MS patients. (DOCX) [file pone.0071058.s002.docx]

**Supplementary Table 1. Correlations between GAB and NSBMS in a group of 58 MS patients**

|  |  | **NSBMS** | | | | | | | |
| --- | --- | --- | --- | --- | --- | --- | --- | --- | --- |
|  |  | PASAT | | SELECTIVE REMINDING | | | 7/24 SPATIAL RECALL | | WORD LIST GENERATION |
|  |  | 3" | 2'' | LTS | CLTR | Delayed | Trials 1-5 | Delayed |  |
| **GAB** | MEMORY | 0.52** | 0.54** | 0.61** | 0.65** | 0.63** | 0.43** | 0.43** | 0.54** |
|  | EXECUTIVE FUNCTION | 0.50** | 0.53** | 0.45** | 0.52** | 0.49** | 0.31* | 0.31* | 0.55** |
|  | VISUAL SPATIAL | 0.32* | 0.47** | 0.41* | 0.45** | 0.39* | 0.35* | 0.12 | 0.33* |
|  | VERBAL FUNCTION | 0.47** | 0.43* | 0.28* | 0.23 | 0.25 | 0.25 | 0.07 | 0.32* |
|  | ATTENTION | 0.39* | 0.49** | 0.42* | 0.50** | 0.51** | 0.27* | 0.45** | 0.62** |
|  | INFORMATION PROCESSING | 0.39* | 0.36* | 0.43* | 0.40* | 0.39* | 0.27 | 0.25 | 0.42* |
|  | MOTOR SKILLS | 0.19 | 0.21 | 0.31* | 0.39* | 0.33* | 0.43* | 0.41* | 0.45* |

LTS: Long-Term Storage

CLTR: Consistent Long-Term Retrieval

***** *p*<0.05

****** *p*<0.001

GAB Visual Spatial, Verbal Function and Motor Skills could not be evaluated as the NSBMS does not include tests in comparable cognitive domains.

Patient mean ± SD age 41.5±10.0 years, disease duration 9.1±7.4 years and EDSS score 2.6 ± 1.8.
