# Supplementary material for: Modeling of Cognitive Impairment by Disease Duration in Multiple Sclerosis: A Cross-Sectional Study
Source: PLoS One. 2013 Aug 1;8(8):e71058. doi: 10.1371/journal.pone.0071058 (PMC3731335; doi:10.1371/journal.pone.0071058)
Supplement: Table S2 — Multiple linear regression analysis: GCS as the dependent variable. (DOC) [file pone.0071058.s003.doc]

**Supplementary Table 2: Multiple linear regression analysis: GCS as the dependent variable**

| Summary of Forward Selection | | | |
| --- | --- | --- | --- |
| Step | Variable Entered | Partial R-Square | Pr > F |
| 1 | EDSS | 0.1416 | <.0001 |
| 2 | Age of onset | 0.0277 | <.0001 |
| 3 | Disease duration | 0.0074 | 0.0003 |
| 4 | Gender | 0.0012 | 0.1492 |
| 5 | MS type | 0.0009 | 0.2023 |
| 6 | IMD Tx | 0.0006 | 0.3077 |
