# Supplementary material for: Modeling of Cognitive Impairment by Disease Duration in Multiple Sclerosis: A Cross-Sectional Study
Source: PLoS One. 2013 Aug 1;8(8):e71058. doi: 10.1371/journal.pone.0071058 (PMC3731335; doi:10.1371/journal.pone.0071058)
Supplement: Table S3 — Odds ratio estimates by logistic regression. (DOC) [file pone.0071058.s004.doc]

**Supplementary Table 3: Odds ratio estimates by logistic regression**

| Analysis of Maximum  Likelihood Estimates  GCS<85 | | | Analysis of Maximum Likelihood Estimates  GCS<70 | |
| --- | --- | --- | --- | --- |
| Variable | Odds ratio  (95%  confidence limits) | p | Odds ratio  (95% confidence limits) | p |
| Disease duration | 1.015  (0.998 – 1.033) | 0.0803 | 1.030 (1.006 – 1.054) | 0.0127 |
| MS type | 1.056  (0.646 – 1.726) | 0.8276 | 0.727 (0.465 – 1.679) | 0.2487 |
| Age of onset | 1.034  (1.021 – 1.048) | <0.0001 | 1.023 (1.006 – 1.040) | 0.0088 |
| Gender | 1.175  (0.905 - 1.526) | 0.2260 | 1.033 (0.730 – 1.462) | 0.8551 |
| EDSS | 0.642  (0.593 – 0.694) | <0.0001 | 0.645 (0.584 – 0.713) | <0.0001 |
| IMD Tx | 1.158  (0.842 – 1.593) | 0.3675 | 1.271 (0.798 – 2.024) | 0.3123 |
