# Supplementary material for: Modeling of Cognitive Impairment by Disease Duration in Multiple Sclerosis: A Cross-Sectional Study
Source: PLoS One. 2013 Aug 1;8(8):e71058. doi: 10.1371/journal.pone.0071058 (PMC3731335; doi:10.1371/journal.pone.0071058)
Supplement: Table S4 — % patients with cognitive impairment at the cutoff level of -1SD and -2SD by disease duration. (DOC) [file pone.0071058.s005.doc]

**Supplementary Table 4: % patients with cognitive impairment at the cutoff**

**level of -1SD and -2SD by disease duration**

| Disease  duration  Years  (number  of patients) | GCS | | Information  Processing  Speed | | Attention | | Verbal  Function | | Visual Spatial  Perception | | Executive  Function | | Memory | | Motor  Skills | | |
| --- | --- | --- | --- | --- | --- | --- | --- | --- | --- | --- | --- | --- | --- | --- | --- | --- | --- |
|  | <70 | <85 | <70 | <85 | <70 | < 85 | <70 | <85 | <70 | <85 | <70 | <85 | <70 | <85 | <70 | <85 |  |
| 1 (187) | 4.3 | 16.6 | 6.0 | 30.8 | 6.4 | 17.1 | 12.0 | 20.7 | 7.1 | 27.2 | 3.8 | 17.8 | 9.7 | 17.3 | 6.6 | 19.8 |  |
| 5 (369) | 6.0 | 20.9 | 9.6 | 34.6 | 6.5 | 20.4 | 12.9 | 23.3 | 12.3 | 22.9 | 6.3 | 25.8 | 9.8 | 20.2 | 7.6 | 21.6 |  |
| 10 (365) | 9.0 | 29.3 | 12.5 | 38.0 | 11.0 | 30.5 | 8.9 | 19.7 | 11.7 | 30.6 | 10.4 | 35.3 | 16.8 | 31.3 | 11.7 | 34.1 |  |
| 15 (245) | 7.8 | 29.0 | 13.5 | 36.5 | 11.2 | 31.8 | 9.9 | 25.0 | 12.3 | 31.2 | 8.3 | 35.0 | 14.5 | 29.5 | 13.0 | 29.0 |  |
| 20 (145) | 10.3 | 33.8 | 16.8 | 37.6 | 17.1 | 32.9 | 14.3 | 22.2 | 12.9 | 25.7 | 12.1 | 34.0 | 16.7 | 34.7 | 14.2 | 33.9 |  |
| 25 (101) | 12.9 | 44.6 | 17.4 | 50.0 | 16.5 | 36.1 | 16.3 | 23.9 | 14.4 | 33.0 | 17.2 | 48.5 | 23.8 | 38.6 | 15.2 | 42.4 |  |
| 30 (88) | 9.1 | 39.8 | 18.4 | 42.1 | 18.4 | 35.6 | 21.3 | 36.3 | 3.5 | 33.3 | 15.1 | 34.9 | 17.2 | 35.6 | 12.0 | 29.3 |  |
